# How Reliable Are YouTube Videos for General Surgery Residents Learning?

**DOI:** 10.7759/cureus.34718

**Published:** 2023-02-07

**Authors:** Tarun Gupta, Tariq H Haidery, Ripudaman Sharma, Sandeep Sharma, Arvind Kumar

**Affiliations:** 1 Surgery, Hamdard Institute of Medical Sciences and Research, New Delhi, IND; 2 College of Medicine, Hamdard Institute of Medical Sciences and Research, New Delhi, IND; 3 Orthopaedics, GS Medical College and Hospital, New Delhi, IND; 4 Neurosurgery, Vardhman Mahavir Medical College and Safdarjung Hospital, New Delhi, IND; 5 Orthopaedics, All India Institute of Medical Sciences, New Delhi, IND

**Keywords:** medical education, videos, general surgery, learning, youtube

## Abstract

Introduction

YouTube is one of the top-searched online video streaming platforms. However, the content of YouTube may not match the standards required for clinical skills learning. Therefore, we investigated the quality of top-viewed YouTube videos related to three basic surgical procedures that need to be performed by general surgery residents in their first year of training in our institute.

Methods

We searched YouTube for the top 10 viewed demonstration videos related to ultrasound-guided abscess drainage, chest tube insertion, and central line insertion. For the eligible videos, we calculated the likes ratio, view ratio, and video power index. The videos’ quality was assessed using LAParoscopic surgery Video Educational GuidelineS (LAP-VEGaS) scores. The videos were categorized into high-quality (LAP-VEGaS score ≥ 11) and low-quality videos (LAP-VEGaS score < 11). The different descriptive (view counts, duration, and likes-dislikes) and calculated parameters stated above were compared between the two video quality-based groups.

Results

The selected videos were uploaded between July 2008 and March 2022. Their mean view counts were 460391.13±373760.19. Their mean video duration was 8.12±4.26 minutes. Their mean likes and dislikes were 2578.38±2977.43 and 144.10±129.80, respectively. The mean like ratio and the mean view ratio were 93.42±13.53 and 317.76±827.79, respectively. The mean video power index was 310.67±827.96. The mean LAP-VEGaS scores for ultrasound-guided abscess drainage, chest tube insertion, and central line insertion-related videos were 6.80, 11.10, and 11.20, respectively. The numbers of likes and dislikes were significantly higher for high-quality videos. Conversely, the view counts, the view ratio, and the video power index were significantly lower for high-quality videos.

Conclusion

Top-viewed videos related to general surgery procedural demonstrations are of low quality. The video view counts, popularity, and likes-dislikes are highly unreliable indicators of surgical video’s usefulness. There is a need for regulatory mechanisms to screen the YouTube content suitable for general surgery residents learning. The residents should therefore be cautious while making inferences based on YouTube videos.

## Introduction

Video-based learning has emerged as one of the preferred teaching-learning modalities in medical education [1-3]. The video-based learning provides a cost-effective alternative when the audience is large; and it is not feasible to provide individualized teaching or teaching in small groups [4]. Moreover, it provides a personalized way of learning in which the students or residents can view and repeat videos conveniently to facilitate better learning. The multiple domains addressed in video-based learning can help in better knowledge retention. For example, the viewers get audio and visual stimulation when watching the video and psychomotor stimulation when they practice and correct themselves based on video steps. The evidence in the literature suggests comparable clinical skills performance among students trained through instructional videos and those trained through live demonstrations [3,5]. Easy availability of instructional videos can help students and residents in better understanding and confidence towards clinical skills [3-5]. In a randomized controlled trial, Buch et al. [6] observed video-based learning to be superior to illustrated text-based e-learning for teaching clinical skills. Fayaz et al. [7] found instructional videos helpful in teaching skills to preclinical students. YouTube is one of the top-searched online video streaming platforms. The freely available content widens its reach to all individuals. Medical students and residents frequently search for clinical videos on YouTube.

Rapp et al. [8] found YouTube as medical students' preferred learning resource for surgical cases. However, it is equally important that the instructional videos be appropriately prepared for teaching-learning purposes. Thus, the videos should be audience-appropriate, tailored, properly structured content, and have a clinical correlation. The procedure details and resolution are equally important. Lengthy videos, poor clinical correlation, and poor resolution can result in losing interest in video-based learning [9]. General surgery residents in our institute often watch clinical skills and surgery-related videos. The widespread use of smartphones and the availability of the internet has made YouTube a preferred platform for online access to clinical videos. However, it needs to be emphasized that the content of YouTube may not match the standards required for clinical skills learning. Likewise, it could result in haphazard and incomplete skills learning among the residents. We hypothesized that the video content on YouTube regarding the basic surgical procedures performed by first-year general surgery residents would not be appropriate for learning and practicing. Therefore, we investigated the quality of top-viewed YouTube videos related to three basic surgical procedures that need to be performed by general surgery residents in their first year of training.

## Materials and methods

On 4^th^ December 2022, we conducted three separate searches on YouTube using the keywords: “ultrasound guided abscess drainage,” “chest tube insertion,” and “central line insertion.” The search results were arranged in decreasing order of view counts. Two faculty members with more than five-year experience in postgraduate teaching of general surgery reviewed the search results. In separate searches, they selected the videos demonstrating the procedural steps of abscess drainage, chest tube insertion, and central line insertion. Any videos without demonstration of procedural steps, non-surgical videos, slideshows or non-video presentations, lectures, webinars, commercial videos, and those unrelated to the searched topics were excluded. A list of the top 10 videos based on view counts was reached for each search separately. We accessed the selected videos’ view counts, age from the upload date, total duration, the number of likes and dislikes, and the nature of the video presenter (whether a medical professional or not). From this information, the likes ratio (total number of likes/sum of likes and dislikes), view ratio (number of views/days since upload), and the video power index (like ratio*view ratio/100) were calculated. In addition, we used the scores calculated using the tool described under LAParoscopic surgery Video Educational GuidelineS (LAP-VEGaS) to assess the quality of reviewed videos [10]. The sub-points specific to laparoscopic procedures and irrelevant to the searched themes were not considered during the assessment. The tool included nine items, with every item scoring from zero (item not presented in the video) to two (item extensively presented in the video) [10]. Thus, the overall assessment scores would range between zero to 18. In our measurements, the continuous variables were expressed as mean±standard deviation, and the categorical variables were expressed as proportions. The videos were then categorized into high-quality (LAP-VEGaS score ≥ 11) and low-quality videos (LAP-VEGaS score < 11). We then compared the view counts, video’s age, total duration, likes ratio, view ratio, and video power index between the low-quality and high-quality videos-based groups. We compared these variables using the Mann-Whitney U test. A p-value of less than 0.05 was considered statistically significant.

## Results

The characteristics of the top 10 selected videos in each of the three categories are shown in Table 1. The selected videos were uploaded between July 2008 to March 2022. Their mean view count was 460391.13±373760.19. Their mean video duration was 8.12±4.26 minutes. Their mean likes and dislikes were 2578.38±2977.43 and 144.10±129.80, respectively. Medical professionals presented all videos except one. The mean like ratio and the mean view ratio were 93.42±13.53 and 317.76±827.79, respectively. The mean video power index was 310.67±827.96. The mean LAP-VEGaS score was 9.70±3.11 ranging between 4 and 15.

**Table 1:** Characteristics of videos that demonstrated procedural steps of different surgical procedures. LAP-VEGaS: LAParoscopic surgery Video Educational GuidelineS

Videos Category	Mean duration (minutes)	Mean View counts	Mean number of likes	Mean number of dislikes	Mean like ratio	Mean view ratio	Mean video power index	Mean LAP-VEGaS score
Ultrasound-guided abscess drainage	5.91	143587.40	327.22	58.86	87.33	51.34	30.25	6.80
Chest tube insertion	7.20	539597.60	2543.44	111.44	93.73	572.08	555.51	11.10
Central line insertion	11.25	697988.40	4265.50	229.81	93.83	329.85	313.95	11.20

Based on LAP-VEGaS scores, there were 14 low-quality and 16 high-quality videos. The comparison of different variables among the two video quality-based groups has been shown in Table 2. The numbers of likes and dislikes were significantly higher for high-quality videos. On the other hand, the view counts, the view ratio, and the video power index were significantly lower for high-quality videos. The duration and like ratio were higher for high-quality videos, but their comparisons between the two groups were statistically insignificant. 

**Table 2:** Comparison between high-quality and low-quality videos based on LAP-VEGaS scores. LAP-VEGaS: LAParoscopic surgery Video Educational GuidelineS

Variable	High-quality videos (LAP-VEGaS score ≥ 11)	Low-quality videos (LAP-VEGaS score ≤ 11)	Statistical significance
Mean duration (minutes)	10.25	5.68	Non-significant, p-value = 0.982
Mean view counts	549673.38	3583542.29	Significant, p-value = 0.026
Mean number of likes	3874.88	982.69	Significant, p-value = 0.001
Mean number of dislikes	188.44	89.54	Significant, p-value = 0.011
Like ratio	94.90	91.58	Significant, p-value = 0.984
View ratio	254.53	390.01	Significant, p-value = 0.032
Video power index	242.74	391.01	Significant, p-value = 0.008

## Discussion

The current analysis’ findings point towards the heterogeneity of video-based learning material available on YouTube. The standard deviations for various parameters were wide, suggesting a large value disparity among the reviewed videos. The content may be appropriate for one procedural skill or theme but may not be the same for others. This disparity is evident from the mean LAP-VEGaS score of ultrasound-guided abscess drainage-related video content, which was too low. Moreover, the content quality of other themes was just borderline. Therefore, the criteria for screening videos based on top view counts may not provide reliable results. The analysis also suggests that it would be hard to differentiate high-quality surgical videos from lower-quality ones merely based on popularity. The lower-quality videos gathered much larger views and had a higher video power index. 

As video-based learning is becoming popular, first-year general surgery residents tend to search for basic surgical content on YouTube. Without regulations, this would result in a constant habit of learning from a source without proven authenticity. Furthermore, unlike the published research and techniques, YouTube videos do not undergo peer review. Moreover, considering their access to the non-medical audience, the popularity indices may reflect something other than the true nature of the information provided in the video. For example, some videos might attract non-medical viewers because of their non-procedural content, which may not help in learning clinical skills. Thus, the curriculum should establish certain norms to prevent trainee residents from gaining knowledge from unregulated YouTube content. Meanwhile, faculty members in surgical disciplines should use online video platforms to present well-structured and high-quality surgical videos, providing better alternatives for trainee residents. Such a strategy would result in more reliable skills learning.

There have been some studies in the past that have assessed the quality of YouTube content related to general and advanced surgical procedures. Savran et al. [11] investigated the effectiveness of YouTube hand surgery videos related to flap reconstruction. While the quality scores contributed by hand specialist-prepared videos were significantly higher, the authors concluded that YouTube videos were unreliable and poorly qualified for hand surgery residents. Regarding hemorrhoid surgery-related videos, Sturiale et al. [12] found that less than one-fourth of the reviewed videos were useful and accurate when assessed by specialists in the field. Anand et al. [13] assessed the quality of the available YouTube videos on pediatric laparoscopic pyeloplasty. The reviewed videos depicted a sub-optimal presentation of the medical information and weak conformity to the LAP-VEGaS guidelines. Luu et al. [14] assessed the quality of YouTube videos available to develop a virtual surgical educational curriculum for trainees in otolaryngology. Very few good-quality videos were found despite having top views. The authors found no correlation between views and video quality. Our analysis supports all these findings. Likewise, a few more studies suggest that medical students/trainees should be cautious while making inferences based on YouTube videos [15,16]. However, the YouTube content for basic surgery-related skills was seldom evaluated.

As far as the videos' likes are concerned, again, the conclusions about the video's educational value are doubtful. It has been suggested the number of likes reflects subjective preference towards the video, which may not be uniform [17]. Kohler et al. [17] observed that the number of likes reflects the popularity of the actors in the video rather than their educational value. Moreover, despite having many likes, it is difficult to confirm the professional status and expertise of the presenters or actors in the videos.

There were some limitations of the current analysis. First, we assessed the quality of surgical videos uploaded on YouTube only. Other video platforms may have a variable impact compared to our findings. However, the freely accessible and widely popular nature of YouTube makes the current analysis worthy. Second, the content was analyzed by general surgery teaching faculty members. While there is a risk of bias in the assessment, we feel the assessment would remain standardized for all videos analyzed in this study. Third, we assessed only the top 10 viewed relevant videos for each category in our analysis. High-quality videos with lower view counts may have been missed. However, that again points towards the heterogeneity of content and its parameters on YouTube. Lastly, we used one particular score for the analysis of videos. Some other tools may quantify videos according to varying attributes. Nevertheless, the LAP-VEGaS tool used in the current analysis is comprehensive and widely used. Thus, variations compared to other tools are likely minor.

## Conclusions

The current study findings suggest that most top-viewed videos related to general surgery procedural demonstrations are of low quality. Moreover, the quality varies with the content searched, and some search themes may generate unreliable results over others. In addition, the video view counts, popularity, and likes-dislikes are highly unreliable indicators of surgical video’s usefulness. Therefore, there is a need for regulatory mechanisms to screen the YouTube content suitable for general surgery residents learning. The residents should therefore be cautious while making inferences based on YouTube videos. In addition, further efforts from surgical disciplines’ faculty members would be needed to create well-structured videos appropriate for educational purposes.
